# Evaluation Frameworks for Clinical Foundation Models in Specific Tasks of Unstructured Medical Text Analysis: A Scoping Review

**DOI:** 10.3390/healthcare14131865

**Published:** 2026-06-26

**Authors:** Laura Johana González Zazueta, Betsaida Lariza López Covarrubias, Christian Xavier Navarro Cota, Mabel Vázquez Briseño, Juan Iván Nieto Hipólito, Gerardo Salvador Romo Cárdenas, Gener J. Avilés Rodríguez

**Affiliations:** 1 Maestría y Doctorado en Ciencias e Ingeniería (MyDCI), Facultad de Ingeniería, Arquitectura y Diseño, Universidad Autónoma de Baja California, Carretera Transpeninsular 3917, Playitas, Ensenada 22860, Baja California, Mexico; lgonzalez85@uabc.edu.mx (L.J.G.Z.); lariza.lopez@uabc.edu.mx (B.L.L.C.); 2 Facultad de Ingeniería, Arquitectura y Diseño, Universidad Autónoma de Baja California, Carretera Transpeninsular 3917, Playitas, Ensenada 22860, Baja California, Mexico; cnavarro@uabc.edu.mx (C.X.N.C.); mabel.vazquez@uabc.edu.mx (M.V.B.); 3 School of Engineering, Andrews University, 8450 E Campus Circle Drive, Berrien Springs, MI 49104, USA; romocardena@andrews.edu; 4 Escuela de Ciencias de la Salud, Universidad Autónoma de Baja California, Carretera Transpeninsular S/N, Valle Dorado, Ensenada 22890, Baja California, Mexico

**Keywords:** clinical foundation models, unstructured medical text, electronic health records, evaluation frameworks, clinical reasoning, ethical and safety considerations

## Abstract

**Background/Objectives:** Clinical foundation models (CFMs) are increasingly being applied to analyze unstructured clinical text, supporting tasks such as clinical reasoning, clinical documentation generation, and information extraction. However, their evaluation remains limited and lacks standardization, which represents a significant challenge for their safe integration into healthcare systems. This scoping review aimed to examine how performance, clinical utility, safety, and generalization are assessed in current evaluation frameworks and methodologies for CFMs applied to electronic health record text and to identify existing gaps and challenges in their evaluation. **Methods:** This scoping review was reported in accordance with PRISMA-ScR guidance, and the study selection process was summarized using a PRISMA flow diagram. A total of 448 records were identified in the initial search, of which 16 met the eligibility criteria and were included in the final synthesis. **Results:** The findings were organized into three categories: methodological frameworks, performance and clinical utility, and ethical and safety considerations. The results show progress in structured methods, evaluation schemes, and simulated environments. Nevertheless, inconsistencies persist in operational robustness, particularly regarding clinical reasoning and hallucinations. The literature also highlights important ethical concerns, including potential biases and regulatory gaps. **Conclusions:** Although CFMs show strong potential to transform clinical text analysis, no comprehensive framework currently guides their evaluation and safe use in real clinical settings. These findings highlight the need for a structured conceptual framework that integrates methodological, technical, clinical, and ethical criteria to support responsible implementation in clinical environments.

## 1. Introduction

### 1.1. General Context and Conceptual Scope: Clinical Foundation Models and Applications

Foundation Models (FMs) are artificial intelligence (AI) systems trained at a large scale on diverse and high-dimensional data. They are characterized by their ability to learn general representations that can be transferred to multiple tasks with minimal adaptation [1]. This ability provides them with considerable flexibility, robustness, and the ability to operate in different domains from their original training setting. When these models are fine-tuned or specialized for healthcare applications, they are known as clinical foundation models (CFMs) [2].

When these models are specialized using biomedical or clinical data, they are referred to in this review as CFMs. These models may integrate biomedical knowledge from large volumes of clinical data such as those found in electronic health records, medical literature, clinical progress notes, or discharge reports. These models are designed to perform multiple tasks in real clinical contexts, such as diagnostic support, assisted documentation, information extraction, or clinical guideline analysis [3].

Large language models (LLMs) are particularly relevant to text-based CFMs because of their ability to understand, generate, summarize, and transform natural language [4,5]. However, not every LLM is considered a CFM, and not every clinical NLP system should be interpreted as a foundation model. Therefore, this review distinguishes between CFMs themselves and clinical applications derived from CFMs. These applications include downstream systems, workflows, benchmarks, or evaluation environments that use FMs or CFMs to perform specific clinical tasks involving unstructured medical text [6,7,8]. Accordingly, the scope of this review includes studies that evaluate CFMs directly, as well as studies that evaluate CFM-based or LLM-based clinical applications when they provide methodological, technical, clinical, safety, ethical, or regulatory evidence relevant to the evaluation of CFMs in unstructured medical text analysis. This distinction is important because the objective of the review is not only to assess model performance, but also to examine how these models and their derived applications are evaluated for clinical utility, safety, generalization, and responsible implementation.

### 1.2. Problem and Importance

The evaluation of CFMs is still limited and inconsistent and therefore methodologically insufficient, despite the increasing interest in and adoption of CFMs. The current literature reports a lack of comprehensive evaluation frameworks that consider clinical, technical and ethical aspects. In addition, many existing studies and evaluation frameworks rely predominantly on a lack of technical metrics disconnected from clinical practice, making it difficult to estimate their true utility in practical scenarios, especially considering the limited consideration of equity, safety and clinical risk fundamental dimensions of responsible implementation [2]. Existing evaluation frameworks still predominantly rely on technical performance metrics that are disconnected from real clinical practice. However, their effective integration into healthcare systems remains fragmented, hindering the effective adoption of CFMs in real clinical environments and highlighting the need to systematically review how they are currently being evaluated.

### 1.3. Objective and Research Questions

The purpose of this review is therefore to critically analyze the existing frameworks, methodologies or approaches for implementing clinical foundation models in real scenarios in order to identify patterns, gaps and pending challenges. such as limited clinical relevance, lack of robust evaluation frameworks and insufficient consideration of ethical and safety aspects. There are three research questions proposed:

1. What frameworks, tools, or methodologies exist to develop or implement clinical foundation models?
2. What is the performance, clinical utility, safety, and generalization capacity of evaluated CFMs?
3. What ethical, regulatory, and institutional challenges are described in the literature on CFMs?

The responses to these research questions connect the choices of methodological design with the clinical, technical and ethical consequences of the implementation of the use of artificial intelligence. It synthesizes the current literature to identify the dominant methodological approaches, evaluation frameworks and current gaps in CFMs assessment. The purpose is to show the evolution of comprehensive and reliable assessment procedures, while keeping clinical relevance, safety, and ethical principles. A structured summary of the problem context, current knowledge, and the specific contributions of this work is presented in Table 1.

## 2. Materials and Methods

### 2.1. Study Design

This section describes three research questions that focus on different aspects of the evaluation of clinical foundation models: development and implementation frameworks; performance evaluation and clinical utility; and ethical, regulatory, and institutional challenges. A meta-analysis was not conducted due to the methodological heterogeneity of the included studies. In addition, no prior review protocol was registered.

A scoping review was conducted to identify, characterize, and analyze the frameworks, methodologies, and approaches used to evaluate clinical foundation models applied to unstructured medical text. Table 2 presents a summary of each research question along with its corresponding motivation.

### 2.2. Information Sources and Search Strategy

This scoping review was conducted and reported in accordance with the Preferred Reporting Items for Systematic Reviews and Meta-Analyses extension for Scoping Reviews (PRISMA-ScR), with reference to the PRISMA 2020 guidance where applicable. No prior review protocol was registered; therefore, no registration code is available [9]. These guidelines supported the identification, evaluation, relevance-based selection, and filtering of studies pertinent to the analysis. The search strategy implemented in the following academic databases: PubMed, Scopus, IEEE Xplore, and Springer Nature. These databases were selected for their strong coverage of multidisciplinary scientific literature. We also included the journal *NEJM AI* due to its emerging relevance in the field of AI applied to healthcare. The terms related to clinical foundation models applied to specific tasks involving unstructured data in electronic health records were combined using Boolean operators. The search was limited to articles published between 2018 and 2025, and only articles published in English language were included. Full details of the searches, including applied filters and structured search strings, are presented in Table 3.

### 2.3. Eligibility Criteria

The study selection process was conducted in two stages. In the first stage, the titles and abstracts of the identified records were screened to exclude those that did not meet the inclusion criteria. Subsequently, the preselected studies were assessed in full text to determine their relevance to the evaluation of CFMs applied to unstructured data contained within electronic health records (EHRs). Studies were included if they met the following criteria:

- Present an evaluation of CFMs applied to unstructured clinical data derived from EHRs.
- Implement or analyze evaluation frameworks, external validation protocols, or comparisons with baseline methods.
- Use de-identified human clinical data with appropriate ethical documentation.
- Report results in English and within the 2018–2025 period, with exceptional inclusion of prior seminal works when highly relevant.

Studies were excluded if they met any of the following criteria:

- Opinion articles, editorials, letters, or commentaries without empirical evidence or verifiable methodology.
- Studies based exclusively on structured data without processing unstructured text.
- Studies involving animal data, synthetic data without real validation, or non-anonymized clinical data.
- Publications in languages other than English or those without a reliable translation.
- Duplicates or multiple versions of the same study; the most complete version was retained.

To preserve conceptual consistency between the research questions, study selection, and scope of the review, eligibility was intentionally focused on studies addressing clinical foundation models, large language models, or applications derived from clinical foundation models applied to unstructured clinical text, particularly in the context of electronic health records, clinical notes, and related medical documentation. Broader concepts related to clinical artificial intelligence, machine learning, or natural language processing were not used as primary search terms because they would have retrieved a large number of studies focused on general clinical AI systems, structured electronic health record data, medical imaging applications, or predictive models outside the scope of this review. Therefore, studies were considered eligible when they provided methodological, technical, clinical, safety, ethical, or regulatory evidence relevant to the evaluation of clinical foundation models in unstructured medical text analysis.

### 2.4. Rationale for Study Inclusion

The unit of analysis in this scoping review was not limited to the foundation model as an isolated computational artifact. Instead, the unit of analysis was defined as the evaluation approach applied to CFMs or to clinical applications derived from CFMs. This decision was based on the objective of the review, which was to examine how CFMs and CFM-based systems are evaluated in the context of unstructured medical text analysis, rather than to compare model architectures alone. Accordingly, studies were included when they provided methodological, technical, clinical, safety, ethical, or regulatory evidence relevant to the evaluation of CFMs or applications based on these models.

This distinction was necessary because many clinically relevant uses of CFMs are evaluated through downstream applications rather than through the foundation model itself. Therefore, studies were considered eligible when their evaluation components were aligned with at least one of the research questions of this review.

### 2.5. Study Selection Process and Data Extraction

**Figure 1:**
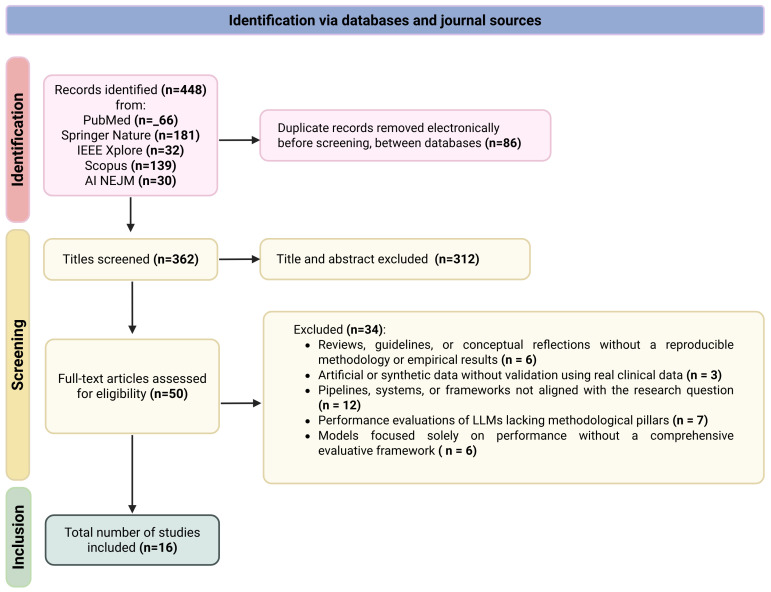
PRISMA flow diagram [10] describing the identification, screening, and eligibility process of the included studies.

A total of 448 records were found in the databases mentioned above. Duplicates were removed (*n* = 86), and 362 studies were screened for eligibility. Of these, 312 were excluded during the title and abstract review because they did not meet the inclusion criteria. Subsequently, 50 articles were retrieved in full text, of which 16 were included in the final synthesis. The entire process is documented in Figure 1, the PRISMA flow diagram [10].

Data extraction was conducted using a predefined extraction matrix. One primary extractor manually extracted information from the full text of each included study, and the extracted information was subsequently reviewed by the research team to identify inconsistencies, missing information, or unclear classifications. The extracted variables included bibliographic metadata; study objective; type of contribution; clinical task; type of data; model or system evaluated; evaluation framework or methodology; evaluation dimensions; reported metrics; use of expert evaluation or external validation; main findings; limitations; and ethical, safety, or governance considerations. When information was unavailable or unclear, it was recorded as “not reported” or “not specified.”

To ensure methodological transparency, we report studies excluded at the final stage of eligibility. Some of these works contained valuable insights on clinical AI, computational workflows, or evaluation strategies, but were not fully compatible with the scope or the research questions proposed in this review. This decision may have reduced the coverage of isolated performance evidence, but it preserved consistency with the objective of identifying evaluation approaches for CFMs and CFM-based applications in unstructured medical text analysis.

The main causes for exclusion were reviews, guidelines or conceptual reflections without reproducible methodology or replicable results (*n* = 6); studies based on artificial or synthetic data without validation by real clinical data (*n* = 3); workflows, systems or frameworks not aligned with the research questions (*n* = 12); studies limited to isolated technical performance metrics without broader evaluative components (*n* = 7); and studies centered on benchmarking or task-specific performance without a broader evaluative framework relevant to CFM implementation (*n* = 6).

Table 4 presents a grouped summary of representative exclusions, including the study reference, its main contribution, and the justification for its exclusion. Benchmark studies were retained only when they aligned with the review’s unit of analysis and research questions by providing evaluation, validation, or implementation evidence for CFMs or CFM-based applications in unstructured clinical text. Benchmarks focused mainly on general medical question answering, examination-style assessment, clinical prediction, or isolated task-level performance were not retained for the final synthesis.

### 2.6. Thematic Classification Procedure and Mapping to the Research Questions

Due to the methodological diversity of the included studies, a thematic classification was conducted to organize the literature according to the type of contribution made. To improve transparency and consistency in the synthesis process, each included study was classified according to its main type of contribution. This classification does not constitute a proposed taxonomy, but rather reflects how the existing literature distributes its approaches around clinical foundation models and their derived applications. Based on the analysis of the studies, three conceptual categories were identified:

1. Methodological frameworks and tools: studies that propose procedures or environments to develop or implement clinical foundation models.
2. Performance evaluation and applicability: investigations that analyze the performance, clinical utility, safety, or generalization capacity of CFMs in specific medical text tasks.
3. Ethical regulations and safety: works that address ethical risks, regulatory considerations, or institutional requirements associated with the use of CFMs in clinical environments.

These categories were used to map the studies to the research questions (RQs) of the review:

1. Category 1 corresponds toRQ1 by identifying existing frameworks and methodologies.
2. Category 2 corresponds to RQ2 by describing how the performance and clinical utility of CFMs are evaluated.
3. Category 3 corresponds to RQ3 by addressing ethical, regulatory, and institutional challenges.

These categories were used to map the included studies to the research questions (RQs) of the review. Category 1 corresponds to RQ1 by identifying existing frameworks, tools, workflows, and implementation methodologies. Category 2 corresponds to RQ2 by describing how the performance, applicability, clinical utility, generalization capacity, and safety related behavior of CFMs are evaluated.Category 3 corresponds to RQ3 by addressing ethical, regulatory, institutional, governance, privacy, bias, and patient safety challenges.

Using the information extracted through the predefined matrix described in Section 2.5, each study was classified according to its primary contribution and mapped to one or more research questions. Because some studies contributed to more than one dimension, multiple RQ assignments were allowed. The initial classification was performed by the primary extractor and subsequently reviewed by the coauthors. Discrepancies or unclear classifications were resolved by consensus after revisiting the full text when necessary.

The final mapping of studies to categories and research questions is presented in Table 5.

## 3. Results

Given that the identified studies address the use of CFMs from multiple perspectives, conceptual criteria commonly found in the literature on AI evaluation in healthcare were used to interpret their contributions in a coherent and comparable manner. This approach also enables comparison among studies with methodological differences, facilitates the identification of patterns and variations in model evaluation, highlights existing gaps, and links the findings to the defined research questions.

**Figure 2:**
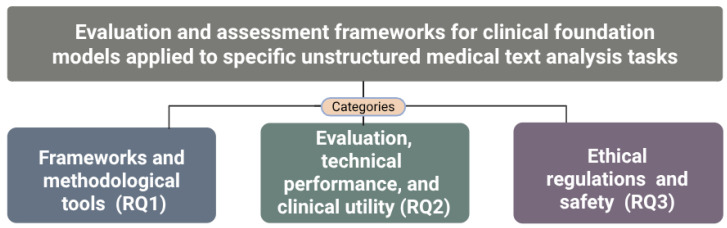
Conceptual structure for the evaluation of clinical foundation models applied to unstructured medical text analysis, organized according to research questions RQ1–RQ3. This figure represents an author-generated conceptual synthesis based on the studies included in this review.

As shown in Figure 2, this structure organizes the studies into three major categories aligned with the research questions: (RQ1) methodological frameworks and tools; (RQ2) evaluation of technical performance, clinical performance, and clinical utility; and (RQ3) ethical regulations and safety considerations. Additionally, six analytical criteria were defined and coded for structured appraisal as follows: C1, methodological frameworks and tools; C2, architectures and operational frameworks; C3, methodological innovation and contribution; C4, performance evaluation and applicability; C5, clinical performance and utility; and C6, ethical regulations and safety, including risks and ethical considerations. This approach is particularly relevant because the included studies not only propose tools and frameworks but also report results related to clinical performance, safety, generalization, reasoning, interaction with simulated and real electronic health records (EHRs), and ethical considerations. Therefore, this structure captures both the common and differential elements among these proposals.

Overall, these criteria allowed the findings to be organized and systematically appraised. In this review, comprehensiveness was not treated as a general descriptive label, but as the explicit coverage of the six analytical criteria defined above. This analytical structure was therefore used as the operational basis for assessing whether any included study could be considered a comprehensive evaluation framework. To systematically assess the extent to which each included study met this operational definition, a structured scoring approach was applied to each criterion: 0 = not addressed, 1 = partially addressed, and 2 = explicitly addressed. A score of 0 was assigned when the criterion was absent; a score of 1 when the criterion was mentioned or indirectly discussed without a specific operational mechanism; and a score of 2 when the study provided an explicit protocol, metric, validation procedure, implementation mechanism, human evaluation process, or governance strategy related to the criterion. This scoring allowed the review to determine whether any included study fulfilled the operational definition of a comprehensive evaluation framework. Table 6 summarizes the studies across six criteria, enabling a comparison of how comprehensive each evaluation approach is.

The structured appraisal shows that none of the included studies explicitly addressed all six criteria for a comprehensive evaluation framework. Although several studies covered methodological, operational, or performance-related dimensions, clinical performance, safety, ethics, and institutional governance were less consistently integrated. Overall, the findings support the review’s central claim that current evaluation approaches remain fragmented, with no included study offering a fully integrated framework for CFMs applied to unstructured clinical text.

### 3.1. Results for RQ1—Existing Frameworks, Tools, and Methodologies

**Figure 3:**
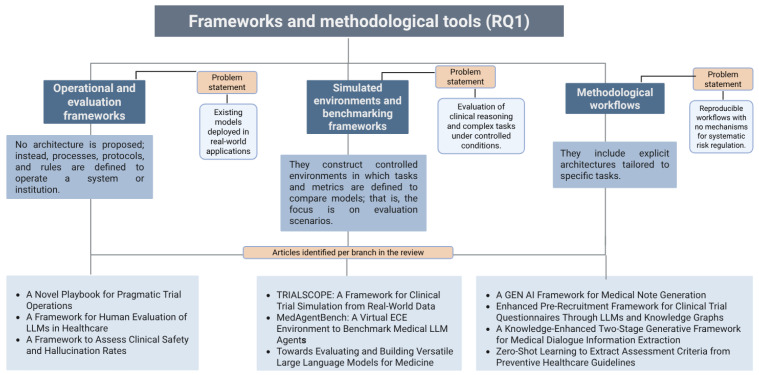
Conceptual representation of the main frameworks, tools, and methodological approaches identified for the development and implementation of clinical foundation models. The figure illustrates how the studies prioritize different components, such as operational architectures, simulated environments, or clinical integration workflows, as well as the key challenges associated with them. This figure represents an author-generated conceptual synthesis based on the studies included in this review [45,46,49,51,52,53,54,55,56,57].

Before examining the specific results related to performance and safety, it was necessary to identify the frameworks, tools, and methodological approaches that support the development and implementation of CFMs in medical text. This step is essential, since it defines the procedures, architectures and operational environments that allow the integration of these models in real clinical scenarios. Detailed information on the studies proposing formalized frameworks, reproducible pipelines, or technical architectures for the operationalization of text-based medical tasks is provided in Supplementary Table S1. The results for RQ1 show that, although the number of methodological frameworks for implementing CFMs in text-based medical tasks, these are distributed across different approaches. Some frameworks prioritize standardized evaluation of performance and safety, others focus on implementation and operational monitoring in clinical environments, and others propose methodological adaptations specific to particular clinical tasks. However, none of the identified approaches fully integrates technical architecture, explicit clinical evaluation, safety, and institutional implementation mechanisms. The splitting of these frameworks hinders their transference to real clinical contexts and evidences a lack of a comprehensive methodological approach for the evaluation of CFMs. To provide an overview of the main methodological approaches identified in the literature, Figure 3 presents a conceptual representation of the frameworks, tools, and operational architectures proposed in this study.

### 3.2. Results for RQ2—Evaluation, Technical Performance, and Clinical Utility

**Figure 4:**
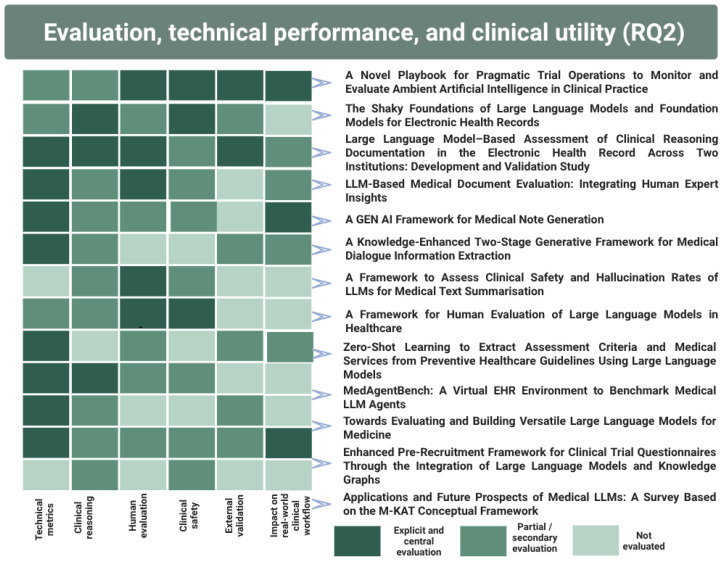
Qualitative heatmap of the evaluation dimensions used in the studies included in RQ2. The color intensity represents the relative frequency with which each dimension is evaluated in the literature, showing a strong concentration on automated technical metrics and a limited, non-systematic evaluation of key dimensions, such as clinical reasoning, safety, external validation, and real-world clinical utility. This figure represents an author-generated conceptual synthesis based on the studies included in this review [45,47,48,49,50,51,52,53,54,55,56,57,58].

Once the methodological frameworks were identified, the analysis focused on the ways in which the studies assessed the technical performance and clinical applicability of CFMs. This category includes studies that not only report metrics, but also put them in the context of real clinical contexts, evaluating aspects such as reasoning, diagnostic accuracy, documentation coherence, safety and utility for healthcare professionals. Detailed information on the studies that conducted empirical, standardized, and clinically relevant evaluations is provided in Supplementary Table S2. These studies enabled the identification of error patterns, limitations, and areas where CFMs demonstrate greater potential. The results for RQ2 show that the evaluation of CFM performance is largely dominated by automated technical metrics that measure precision, textual similarity, or functional accuracy, but that capture real clinical utility only to a limited extent. Although many studies incorporate human evaluation, analysis of clinical reasoning, or explicit measurement of risks such as hallucinations, these approaches remain exceptional and are not applied in systematic way, often being mentioned only as considerations in the selected studies. Overall, current evaluations of CFMs make it difficult to interpret their results as sufficient evidence for decision making in real clinical environments. To comparatively visualize the dimensions used in evaluating performance and clinical utility of clinical foundation models, Figure 4 presents a qualitative heatmap summarizing the evaluative criteria employed in the studies included in RQ2.

### 3.3. Results for RQ3—Ethics and Institutional Safety

**Figure 5:**
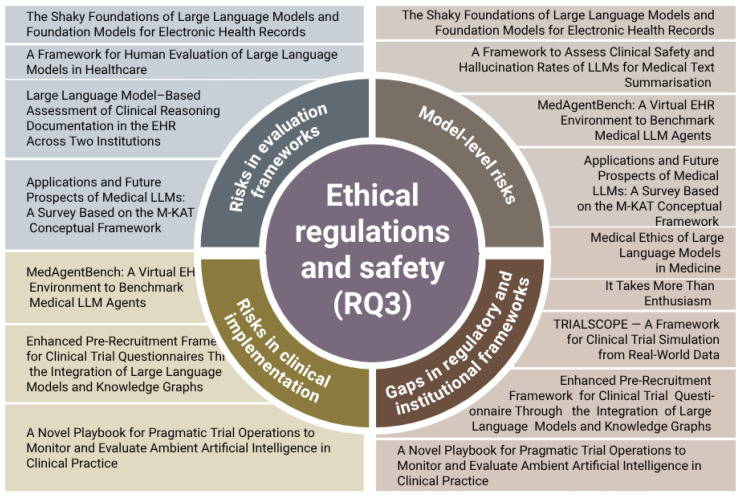
Synthesis of the main ethical, safety, and institutional governance risks associated with the use of clinical foundation models. The figure reflects which of the included studies address each category of risk, organizing them from the model level to clinical implementation and regulatory frameworks, and highlighting their cumulative nature as well as the absence of an integrated institutional framework for mitigation. This figure represents an author-generated conceptual synthesis based on the studies included in this review [45,46,47,48,49,52,54,55,58,59,60].

Finally, the contributions addressing ethical aspects, risks, and clinical safety were analyzed, as these elements are essential for the responsible adoption of CFMs in real-world environments. Unlike technical performance, this category focuses on risks derived from clinical errors, biases, insufficient human supervision, and the lack of regulatory infrastructure. Detailed information on the studies that explicitly discuss ethical, regulatory, and institutional safety considerations is provided in Supplementary Table S3. The results for RQ3 show that the reviewed studies consistently identify ethical and safety risks associated with the use of CFMs, particularly those related to clinical hallucinations, biases affecting underrepresented populations, lack of traceability in model decisions, and limited human oversight. The literature also points to recurring deficiencies in regulatory mechanisms, institutional control structures and standardized procedures for the safe adoption of CFMs. More generally, these results indicate that ethical and safety issues are treated in a fragmented and heterogeneous way, without a common institutional framework articulating technical evaluation, clinical responsibility and operational regulatory structures. To synthesize the main ethical, safety and regulatory issues discussed in the literature, Figure 5 conceptually organizes the categories of risk identified in the included studies.

## 4. Discussion

### 4.1. Synthesis of Findings

This review shows that the literature on clinical foundation models (CFMs) applied to unstructured medical text has progressed significantly across three major categories, ranging from the design of frameworks and operational environments for their development and implementation to proposals for evaluating performance and clinical utility, and an increasing recognition of ethical, regulatory, and institutional risks. Despite these advances, the central finding is that they remain dispersed, as most studies focus in depth on one or two categories, but few simultaneously integrate how models are implemented (RQ1), how they are evaluated with clinical relevance (RQ2), and how risks are regulated and mitigated in real clinical settings (RQ3). This fragmentation occurs because technical, clinical, ethical, and regulatory studies usually focus on different aspects and use different methods. For this reason, current approaches are hard to compare and rarely show a complete path from model development to safe clinical use. Such fragmentation helps explain why, despite promising results, the adoption of CFMs remains uneven and difficult to compare across clinical contexts according to the existing literature.

**Figure 6:**
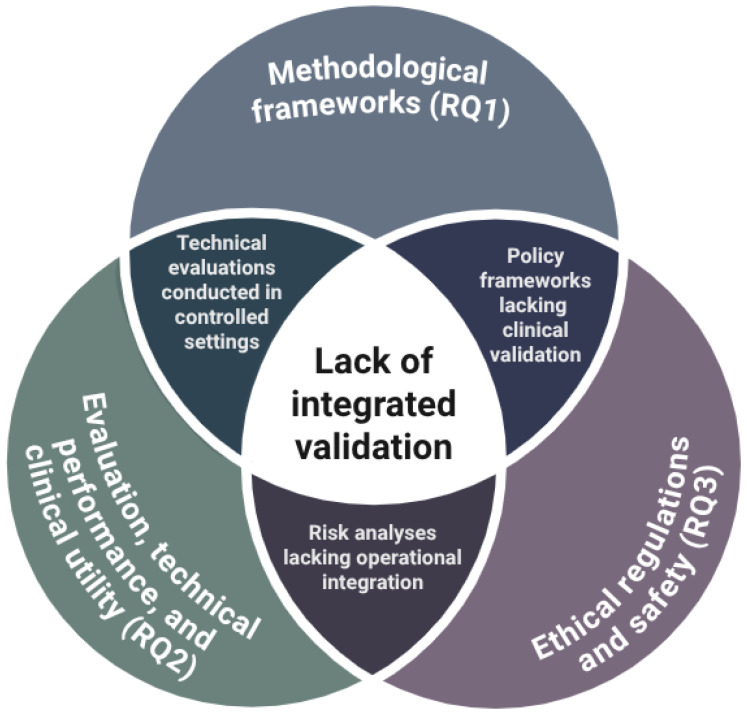
Graphical representation of the fragmented way in which the literature on clinical foundation models (CFMs) addresses the dimensions of implementation, clinical utility evaluation, and ethical and safety regulation. It can be observed that most studies deal with only one or two of these categories, which limits the comparability of results and the responsible adoption in real clinical settings. This figure represents an author-generated conceptual synthesis based on the studies included in this review.

The main finding of this review is the lack of studies that address these three categories in an integrated way, which hampers comparison of results and complicates the responsible implementation of these models in real clinical settings. In summary, the information collected support a single major conclusion: clinical utility cannot be inferred from quantitative measures alone. The included studies agree that CFMs should be evaluated in light of medical reasoning, explicit safety mechanisms, and appropriate institutional conditions to be considered responsibly implementable. Figure 6 does not aim to describe a development workflow, but rather to illustrate the structural disconnection among the main domains analyzed in this review.

### 4.2. Results by Research Question

**Figure 7:**
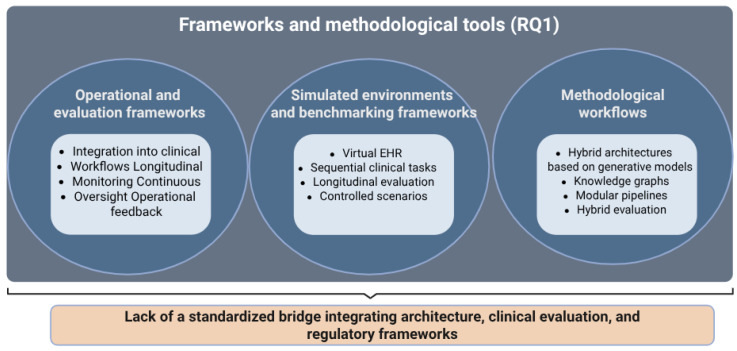
Summary of the main methodological frameworks and tools identified in RQ1. The figure categorizes the proposals into three groups: operational frameworks for clinical deployment and continuous monitoring, simulated environments for testing challenging clinical tasks, and methodological workflows based on hybrid architectures. In summary, the absence of a standardized framework that integrates architecture, clinical evaluation and regulation is highlighted. This figure represents an author-generated conceptual synthesis based on the studies included in this review.

The results concerning RQ1 reveal a variety of methodological proposals that can be grouped into three main patterns, summarized in Figure 7. The first are operational frameworks that are focused on deployment and monitoring in the real world. Works like Afshar et al. [45] emphasize the need to integrate models into existing clinical workflows and to develop processes for continuous monitoring and feedback. These investigations reflect a shift in focus, prioritizing long-term viability and system integrity rather than relying solely on immediate accuracy outcomes.

Second, a group of studies, such as those by Jiang et al. [49], focus on evaluating complex clinical tasks within controlled environments. This approach is especially relevant for clinical text, where tasks are often sequential and context-dependent, and sensitive to the patient history conditions rarely captured by isolated point evaluations.

Finally, applied frameworks merge CFMs with special purpose modules. Frameworks that combine components such as ASR, RAG, LLMs, and knowledge graphs are provided in works such as those by Leong et al. [51] and Zihang et al. [52], aiming to integrate these systems into real clinical workflows for purposes such as documentation or recruitment. At the same time, proposals like those by Seo et al. [50], with hybrid expert-based evaluation, and Tam et al. [54], with structured human evaluation frameworks, formalize reproducible evaluation processes.

In conclusion, the results of RQ1 suggest a strong methodological foundation for the realization of CFMs; however, this basis is fragmented, with some approaches tending to integrate in real-world settings, others to controlled simulations and others to methodological workflows. A common bridge between architecture, clinical evaluation and regulatory frameworks is still to be established.

**Figure 8:**
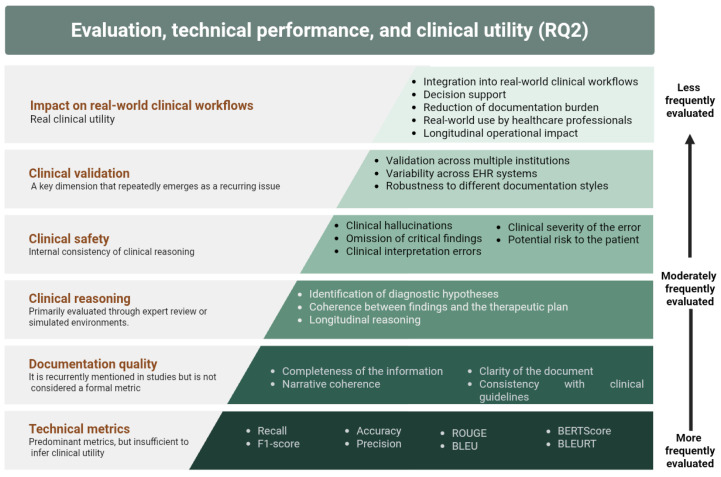
This figure summarizes the metrics and criteria reported in the studies included in RQ2. The intensity of the color represents the relative frequency with which each dimension is examined in the literature. There is a strong focus on automated technical metrics assessed at high frequency and a limited and non-systematic evaluation of crucial dimensions such as clinical reasoning, safety, external validation and clinical utility in real-life situations. This figure represents an author-generated conceptual synthesis based on the studies included in this review.

The results obtained for RQ2 describe an evolutionary trend from technical metrics to evaluation strategies more sensitive to the clinical context. As shown in Figure 8, the literature is biased toward automated technical metrics, whereas critical dimensions such as clinical reasoning, safety, multi-institutional validation and actual clinical utility are evaluated in a limited and non-systematic manner. Although metrics such as ROUGE, BERTScore and BLEURT [18] are still widely used in many studies, these works acknowledge their limitations in capturing important aspects such as clinical reasoning, completeness of information, narrative coherence and the risk of hallucinations.

Based on the analysis provided, it is possible to conclude that there are some key points that can be identified. Performance can be unstable across distribution shifts, differences in institutional EHR systems, or variations in documentation styles, as demonstrated by Wornow et al. [47] and others. This limits generalizability and highlights the need for external validation and multi-center assessment.

Multiple studies have reported promising results in terms of performance. For example, works that introduced task-specific benchmarks for medical texts, e.g., work by Wu et al. [57], show that CFMs consistently outperform previously specialized models across a range of tasks, but with substantial variability in complex clinical tasks or low-shot instruction settings. In addition, evaluations in simulated EHR environments, such as that proposed by Jiang et al. [49], show that LLM-based agents can successfully perform longitudinal clinical tasks, especially in simulated settings. However, other studies suggest that performance is sensitive to task complexity and the degree of environmental structuring, as noted by Hu et al. [53] and Ong et al. [59].

In terms of clinical applicability, the literature suggests that models are more promising when integrated into specific task-oriented workflows rather than evaluated as stand-alone systems. For example, Leong et al. [51] suggest that models could improve the efficiency and quality of clinical documentation, but only if used as a supportive tool under human supervision. Likewise, in another study, Zihang et al. [52] reported that the identification of candidates for clinical trials has been enhanced, but expert validation is required to avoid inappropriate exclusion. These findings underscore the need to evaluate model performance not only by technical metrics but also by its practical impact on documentation, decision making, and clinical efficiency.

**Figure 9:**
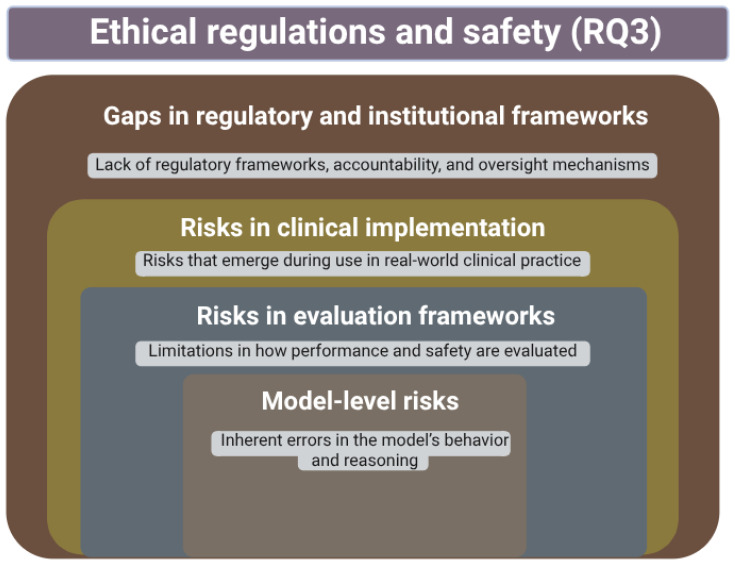
Risk map for the layered adoption of CFMs. Risks are multilevel, as shown in the diagram, and they originate from model behavior and reasoning, are conditioned by limitations in evaluation frameworks, are manifest during clinical implementation, and are amplified in the absence of adequate regulatory and institutional frameworks. This figure represents an author-generated conceptual synthesis based on the studies included in this review.

In contrast, the studies on RQ3 agree that ethical and regulatory challenges are one of the main barriers for the adoption of CFMs in clinical practice. In this work, these challenges are organized with a layered risk map that shows how risks are generated at different levels and associated across the model lifecycle as shown in Figure 9. In addition to ethical and safety concerns, the clinical deployment of CFMs must comply with health data protection regulations, such as HIPAA [61], GDPR [62], and KVKK [63]. These frameworks impose requirements on privacy, lawful data processing, data sharing, and institutional accountability, highlighting the need for legal-readiness criteria before clinical deployment.

First, clinical risk and patient safety appear as central axes of discussion in most articles in this category. One particularly noteworthy observation is that analyses of hallucinations in clinical summaries show that generative errors can be clinically dangerous even when they are linguistically coherent, emphasizing the necessity of systematic auditing mechanisms and continuous monitoring, as pointed out by Asgari et al. [55] and Wornow et al. [47].

Secondly, several studies demonstrate the lack of sufficient institutional infrastructure to support regulatory mechanisms for these models, as noted by Turner et al. [60]. Afshar et al. [45] indicate the importance of setting out clear procedures for human oversight, institutional responsibility and longitudinal monitoring when deploying such models in the clinical setting. Similarly, Tam et al. [54] emphasize the importance of structured human evaluation as a key component of effective regulation.

Additionally, it raises issues of impartiality, bias, and professional responsibility, particularly in applications influencing clinical decision making or patient selection and prioritization processes [52].

As a general pattern, the results of RQ3 suggest that ethics and regulation should not be a secondary consideration, but rather a consideration that is integrated into the early stages of design, evaluation and implementation of CFMs. The challenges identified in RQ3 are consistent with broader health AI governance frameworks, which emphasize risk management, transparency, accountability, human oversight, and post-deployment monitoring. However, these frameworks were not included as studies because this review focused on evaluation approaches for CFMs applied to unstructured clinical text. Their relevance is that they highlight the need to translate high-level governance principles into operational, task-specific evaluation criteria for real clinical settings. Bias-related findings were analyzed only according to what was explicitly reported in the included studies. This review did not independently assess demographic bias, prompt sensitivity, distributional shift, or dataset representativeness. Overall, these risks were addressed inconsistently and rarely through standardized metrics, subgroup analyses, prompt variation tests, or external validation. This reinforces the need for standardized criteria to evaluate fairness, robustness, and generalizability of CFMs in clinically meaningful contexts.

### 4.3. Studies Integrating RQ1, RQ2, and RQ3

**Figure 10:**
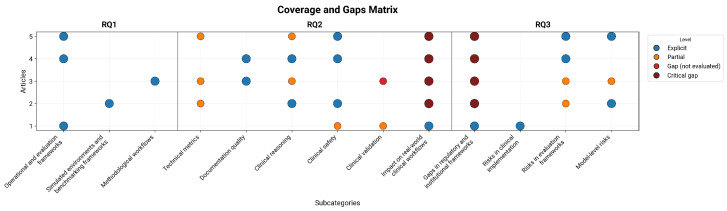
The diagram shows the coverage of subcategories related to the three research axes (methodology for implementation, performance in the clinic, and considerations of ethics/regulation) across the five main studies. This graphic indicates the uneven coverage of the three main aspects of the research in each study and highlights their strengths, as well as the aspects to be covered more in future studies.

This review shows that the main finding is that only a few studies address methodological aspects of implementation (RQ1); performance and clinical applicability (RQ2); and ethical, regulatory and institutional considerations (RQ3) at the same time. This pattern is clearly visible in Figure 10, which shows a dot matrix of the five reviewed studies that cover these key areas. The diagram’s colors represent the coverage level in each subcategory: blue indicates explicit coverage, orange indicates partial mention, red indicates not evaluated, and dark red indicates critical gaps identified in the literature review.

The matrix indicates that, in general, studies address fully or partially only some of these research axes, without a full integration of all three key dimensions. Work by Afshar et al. [45] is significant as it incorporates the assessment of model performance directly in real clinical use environments. The main contribution of this approach, as shown in the corresponding table, is to demonstrate that the clinical utility of artificial intelligence cannot be assessed in a static way, but must be understood as a dynamic process that changes in relation to workflows and institutional conditions.

Jiang et al. [49] represent a significant methodological advancement by providing a virtual EHR environment for evaluating LLM-based clinical agents. Zihang et al. [52] stand out for their hybrid approach, integrating generative models with knowledge graphs to evaluate clinical applicability within a highly regulated process such as clinical trial recruitment. As demonstrated in the mapping tables, this study aims to clearly show how architectural decisions can influence ethical and regulatory risks. The main contribution of Tam et al. [54] is the standardization of human evaluation by clinically relevant criteria, i.e., quality, utility, applicability, and safety, with a methodological evaluation framework in agreement with the judgment of healthcare professionals and ethical regulations. Finally, the work in Asgari et al. [55] is notable for turning clinical safety into an evaluable and measurable construct, by providing a concrete tool to assess risks in generative tasks by explicitly accounting for the potential impact of errors, rather than limiting the evaluation to textual similarity metrics.

In conclusion, the five studies provide a strong conceptual and methodological foundation to support the development and implementation of CFMs, although each deals with the problem from a complementary, but not unified, perspective. This diversity of approaches is not a weakness of the existing literature, but rather an expression of the systemic complexity associated with the use of CFMs in health care environments. However, the dot matrix clearly demonstrates the absence of an integrative framework that can systematically articulate architectural decisions, clinical evaluation schemes and institutional regulatory mechanisms. In this context, a structured taxonomy is needed to organize these approaches, identify clear methodological pathways, and guide selection and implementation depending on the type of clinical task, risk level and institutional context.

### 4.4. Key Evaluation Components Identified Across the Included Studies

To further operationalize the synthesis presented in this review, Table 7 summarizes the main evaluation components identified across the included studies, organized according to the three major categories aligned with the research questions and the six analytical criteria used in the analysis.

This table shows that the included studies cover the three major categories proposed in this review and provide evidence across the six analytical criteria. However, these components are not integrated consistently within a single evaluation approach. Most studies emphasize one or two dimensions, such as technical performance, clinical applicability, or safety, while fewer studies connect methodological design, clinical validation, and ethical or institutional governance. This fragmentation supports the need for more comprehensive and operational evaluation frameworks for CFMs applied to unstructured clinical text.

### 4.5. Limitations

This scoping review has several limitations. The search was restricted to a limited set of databases and sources and included only English-language publications, which may have excluded relevant studies in other languages, gray literature, preprints, institutional reports, or regulatory documents. In addition, the field of clinical foundation models is evolving rapidly, so the findings may change as new models, evaluation methods, deployment strategies, and regulatory frameworks emerge.

Another important limitation is the variability of terminology in this area, as relevant studies may use alternative terms such as medical language models, EHR foundation models, clinical NLP, ambient AI, or downstream clinical applications. Potential publication bias should also be considered, as published literature may favor positive results and successful implementations while underreporting failures, safety risks, or operational barriers. Finally, no prior protocol was registered, which may limit reproducibility, although the study selection and data extraction criteria were transparently documented.

## 5. Conclusions

Although there has been rapid progress in the development and application of CFMs for the analysis of unstructured medical text, this review reveals an implicit gap in the evaluation of these models. The existing literature describes a variety of frameworks, metrics and approaches that partly cover technical performance, clinical applicability, or ethical risks, but these contributions are fragmented and not systematically integrated to inform their responsible adoption in real clinical environments.

The major discrepancy identified is not the lack of evaluation methods, but the absence of an integrative framework to coherently link methodological decisions; clinically relevant performance criteria; and institutional safety, ethical and regulatory mechanisms. Such a lack of integration limits the comparability of studies, complicates external validation, and prevents the translation of experimental results into clinical practice.

According to the analysis captured in this review, we identify three pillars we consider fundamental and propose as minimum elements for future work with the objective of evaluating applied CFMs. The first pillar is methodological frameworks and tools that define how models are developed, integrated and operated in real-world contexts. The second pillar is criteria for performance and clinical utility evaluation, including reasoning, safety and clinical impact, in addition to technical metrics. The third pillar is consideration of ethical, safety, and institutional regulatory elements for reducing risks and encouraging responsible implementation.

The findings of this work suggest that, rather than proposing a single implementation pathway or a definitive evaluative framework, the nature of the challenge calls first for the construction of conceptual foundations that may eventually enable actionable strategies for meaningful implementation in clinical environments. In practical terms, the findings of this review provide a structured foundation that can support researchers, clinicians, and healthcare institutions in selecting, evaluating, and implementing clinical foundation models in real-world settings. By identifying key methodological, clinical, and ethical dimensions, this work contributes to more informed decision-making processes and promotes the development of safer, more reliable, and clinically meaningful AI systems in healthcare. As future work, we propose the development of a structured categorization that integrates these three pillars as foundational elements, enabling the classification of existing approaches; the implementation of analytical levels according to clinical task type, risk level, and institutional context; and the facilitation of more balanced, comparable, and feasible evaluations. The construction of such a categorization would represent a step toward coherent, global, and clinically meaningful validation strategies that support real-world application, thereby contributing to closing the existing gap between innovation in clinical foundation models and their safe adoption in healthcare systems.

## Figures and Tables

**Table 1:** Statement of significance.

Problem or issue	Insufficient and non-standardized evaluation of CFMs in unstructured clinical text processing tasks.
What is already known	CFMs, particularly those based on large language models, have shown strong performance in clinical tasks such as information extraction, summarization, and decision support; however, their evaluation remains inconsistent, primarily focused on technical metrics with limited integration of clinical, ethical, and safety considerations.
What this paper adds	This paper critically reviews existing evaluation frameworks, identifies methodological gaps, and proposes a structured perspective that integrates technical, clinical, and ethical dimensions, promoting more comprehensive and context-aware evaluations.
Who benefits from this knowledge	Clinical AI researchers, healthcare professionals, model developers, policymakers, and decision makers interested in the safe and effective implementation of CFMs.

**Table 2:** Research questions (RQs) and motivation for the review.

Research Question (RQ)	Motivation
RQ1: What frameworks, tools, or methodologies exist to develop or implement clinical foundation models?	Identify which approaches have been proposed to build, integrate, or operationalize CFMs in clinical tasks and how these frameworks guide their current implementation.
RQ2: What is the performance, clinical utility, safety, and generalization capacity of CFMs evaluated?	Explore the methods and metrics used to evaluate CFMs, determining whether they truly capture clinical performance, safety, and robustness across different contexts.
RQ3: What ethical, regulatory, and institutional challenges are described in the literature on CFMs?	Analyze the risks, barriers, and institutional requirements that must be considered to implement CFMs responsibly and safely in real clinical environments.

**Table 3:** Databases, search strings, publication years of retrieved records, and number of identified records.

Database and Publication Years of Retrieved Records	Search String	Results
PUBMED (2023–2025)	(“clinical foundation model * ” OR “large language model *” OR “Clinical Foundation Models”) AND (“electronic health record *” OR “EHR” OR “clinical notes”) AND (“evaluation framework” OR “benchmark *” OR “assessment”)	66
IEEE Xplore (2024–2025)	(“clinical foundation model *” OR “large language model *” OR “Clinical Foundation Models”) AND (“electronic health record *” OR “EHR” OR “clinical notes”) AND (“evaluation framework” OR “benchmark *” OR “assessment”)	32
SCOPUS (2023–2025)	(“clinical foundation model *” OR “large language model *” OR “Clinical Foundation Models”) AND (“electronic health record *” OR “EHR” OR “clinical notes”) AND (“evaluation framework” OR “benchmark *” OR “assessment”)	139
SPRINGER NATURE (2018–2025)	(“clinical foundation model *” OR “large language model *” OR “Clinical Foundation Models”) AND (“electronic health record *” OR “EHR” OR “clinical notes”) AND (“evaluation framework” OR “benchmark *” OR “assessment”) AND (“unstructured data” OR “clinical text” OR “free text” OR “narrative note *”)	181
NEJM AI (2018–2025)	(“clinical foundation model *” OR “large language model *” OR “Clinical Foundation Models”) AND (“electronic health record *” OR “EHR” OR “clinical notes”) AND (“evaluation framework” OR “benchmark *” OR “assessment”)	30

Note. The search was conducted using the 2018–2025 time window across all databases. The years shown indicate the publication years of the records retrieved from each database, not different database-specific search limits. The Springer Nature search string included additional terms related to unstructured clinical text (“unstructured data” OR “clinical text” OR “free text” OR “narrative note *”) to increase specificity due to the high volume and broad scope of results in that database; these terms were aligned with the focus of this review on unstructured medical text.

**Table 4:** Excluded studies at the final stage of eligibility and justification for exclusion. The table groups representative works excluded from the final analysis, indicating their main contribution and the methodological or conceptual reasons for exclusion according to the defined criteria.

Exclusion Category	Articles	Main Contribution	Justification for Exclusion
1. Reviews, guidelines, or conceptual reflections without replicable methodology (*n* = 6)	[11,12,13,14,15,16]	Critical or reflective reviews on AI, biases, regulations, or theoretical methods.	Do not present experiments, metrics, clinical datasets, or replicable methodology.
2. Studies based exclusively on synthetic data (*n* = 3)	[17,18,19]	Evaluations or methods applied using simulated or synthetic data.	Lack subsequent validation with real clinical data or expert-annotated datasets.
3. Pipelines, systems, or frameworks not aligned with the research questions (*n* = 12)	[20,21,22,23,24,25,26,27,28,29,30,31]	Systems, tools, or workflows designed for specific tasks (Named Entity Recognition (NER), matching, summarization, prediction, U-health).	Technical applications that do not develop a general evaluative methodological framework for CFMs.
4. Studies limited to isolated technical performance metrics without broader evaluative components (*n* = 7)	[32,33,34,35,36,37,38]	Model performance evaluations in clinical tasks such as extraction, classification, coding, or prediction.	Report useful technical performance results, but do not include broader evaluation components related to clinical utility, safety, robustness, implementation, or governance.
5. Studies centered on benchmarking or task-specific performance without a broader evaluative framework (*n* = 6)	[39,40,41,42,43,44]	Benchmarks and model comparisons focused on task-level performance or technical metrics.	Do not incorporate a broader evaluative framework aligned with clinical validation, safety assessment, implementation, or responsible CFM evaluation.

**Table 5:** Mapping of the included studies according to their contribution to the research questions (RQ1–RQ3) and their primary type of contribution. The symbol “√” indicates that the criterion was addressed in the corresponding study.

Article	Primary Type of Contribution	RQ1	RQ2	RQ3
A Novel Playbook for Pragmatic Trial Operations to Monitor and Evaluate Ambient Artificial Intelligence in Clinical Practice [45]	Real-world clinical implementation, monitoring, and governance framework	√	√	√
TRIALSCOPE—A Framework for Clinical Trial Simulation from Real-World Data [46]	Clinical trial simulation and validation framework using real-world data	√		√
The Shaky Foundations of Large Language Models and Foundation Models for Electronic Health Records [47]	Critical evaluation of reliability, robustness, and safety limitations in EHR-based FMs/LLMs		√	√
Large Language Model-Based Assessment of Clinical Reasoning Documentation in the EHR Across Two Institutions [48]	Multi-institutional assessment of clinical reasoning and documentation quality		√	√
MedAgentBench: A Virtual EHR Environment to Benchmark Medical LLM Agents [49]	Virtual EHR benchmark for evaluating medical LLM agents	√	√	√
LLM-Based Medical Document Evaluation: Integrating Human Expert Insights [50]	Hybrid automated and expert-informed medical document evaluation framework	√	√	
A GEN AI Framework for Medical Note Generation [51]	Generative AI workflow for clinical note generation	√	√	
Enhanced Pre-Recruitment Framework for Clinical Trial Questionnaires Through LLMs and Knowledge Graphs [52]	LLM- and knowledge graph-based workflow for clinical trial pre-recruitment	√	√	√
A Knowledge-Enhanced Two-Stage Generative Framework for Medical Dialogue Information Extraction [53]	Knowledge-enhanced information extraction framework for medical dialogues	√	√	
A Framework for Human Evaluation of Large Language Models in Healthcare [54]	Human evaluation framework for healthcare LLM outputs	√	√	√
A Framework to Assess Clinical Safety and Hallucination Rates of LLMs for Medical Text Summarisation [55]	Clinical safety and hallucination assessment framework	√	√	√
Zero-Shot Learning to Extract Assessment Criteria from Preventive Healthcare Guidelines [56]	Zero-shot extraction framework for guideline-derived clinical criteria	√	√	
Towards Evaluating and Building Versatile Large Language Models for Medicine [57]	Multitask benchmark and evaluation framework for medical LLMs	√	√	
Applications and Future Prospects of Medical LLMs: A Survey Based on the M-KAT Conceptual Framework [58]	M-KAT conceptual framework for mapping medical LLM applications, capabilities, and risks		√	√
Medical Ethics of Large Language Models in Medicine [59]	Ethical and regulatory analysis of LLM use in medicine			√
It Takes More Than Enthusiasm: The Missing Infrastructure to Unlock AI’s Potential in Medical Education [60]	Institutional governance and infrastructure analysis for AI implementation			√

**Table 6:** Structured appraisal of included studies according to operational criteria for comprehensive evaluation.

Included Study	C1	C2	C3	C4	C5	C6	Coverage Score
[45]	2	2	1	1	2	2	10
[46]	2	2	2	2	1	0	9
[47]	0	0	1	1	1	2	5
[48]	1	0	1	1	2	1	6
[49]	2	2	2	2	1	1	10
[50]	2	1	2	2	2	0	9
[51]	1	2	1	2	1	0	7
[52]	1	2	2	2	1	1	9
[53]	1	2	2	2	1	0	8
[54]	2	1	2	1	2	2	10
[55]	2	1	2	2	1	2	10
[56]	1	1	2	2	1	0	7
[57]	2	1	2	2	1	0	8
[58]	1	0	1	1	1	2	6
[59]	0	0	1	0	0	2	3
[60]	0	1	1	0	0	2	4

**Table 7:** Summary of the evaluation frameworks for clinical foundation models identified in the included studies.

RQ	Analytical Criterion and Operational Components	Representative Studies	Main Synthesis
RQ1	Methodological frameworks and tools: evaluation protocol, reproducible workflow, data extraction strategy, validation procedure, and human review process.	[45,46,49,50,54,55,57]	Structured frameworks are proposed, but they are usually task-specific.
RQ1	Architectures and operational frameworks: EHR integration, simulated EHR environments, ASR, RAG, knowledge graphs, LLM agents, and monitoring pipelines.	[45,49,51,52,53]	Operational architectures are described, but are not consistently linked to clinical validation or governance.
RQ1	Methodological innovation and contribution: hybrid evaluation, expert-informed assessment, zero-shot extraction, clinical trial simulation, and longitudinal monitoring.	[46,50,52,54,55,56]	Innovative approaches are evident, but remain heterogeneous and difficult to compare.
RQ2	Performance evaluation and applicability: accuracy, precision, recall, F1-score, ROUGE, BLEU, BERTScore, extraction accuracy, and classification performance.	[49,50,51,52,53,56,57]	Technical metrics are frequently reported, but are insufficient to determine clinical usefulness alone.
RQ2	Clinical performance: clinical reasoning, documentation quality, completeness, coherence, guideline concordance, clinician review, and external validation.	[45,48,49,50,51,54,58]	Clinical performance is increasingly considered, but without standardized criteria across studies.
RQ3	Ethical regulations and safety: hallucination rate, omission and distortion errors, potential patient harm, bias, fairness, transparency, accountability, and oversight.	[45,47,49,52,54,55,58,59,60]	Safety, ethics, and governance are recognized as essential, but remain fragmented and non-systematic.

## Data Availability

No new datasets were generated during this scoping review. The data supporting the reported results were derived from the published studies included in the review and are available within this article, its tables, and the cited publications. The review protocol is available in the Open Science Framework repository [64].

## Supplementary Materials

The following supporting information can be downloaded at: https://www.mdpi.com/article/10.3390/healthcare14131865/s1. Table S1: Studies proposing frameworks, tools, or methodologies for the development and implementation of clinical foundation models (RQ1); Table S2: Studies evaluating technical performance and clinical applicability of clinical foundation models (RQ2); Table S3: Studies addressing ethical, regulatory, and institutional safety considerations of clinical foundation models (RQ3).
