# Supplementary material for: Evaluation Frameworks for Clinical Foundation Models in Specific Tasks of Unstructured Medical Text Analysis: A Scoping Review
Source: Healthcare (Basel). 2026 Jun 26;14(13):1865. doi: 10.3390/healthcare14131865 (PMC13360617; doi:10.3390/healthcare14131865)
Supplement: Supplementary file 1 [file healthcare-14-01865-s001.zip › healthcare-4350993-supplementary.pdf]

Supplementary Materials

Table S1: Studies proposing frameworks, tools, or methodologies for the development and implementation of clinical foundation models (RQ1)

Full Article Title	Year	Reason for Inclusion	Architectures and Operational Frameworks	Innovation and Methodological Contribution
A Novel Playbook for Pragmatic Trial Operations to Monitor and Evaluate Ambient Artificial Intelligence in Clinical Practice [1]	2024	Proposes a formal, replicable playbook oriented toward real clinical practice within hospital environments.	Operational framework composed of five domains: governance, clinical user experience, technical integration with EHR, documentation and traceability processes, and performance analytics with longitudinal monitoring.	Standardizes AI adoption and monitoring processes in clinical environments; incorporates continuous feedback cycles, safety indicators, and clinical usage metrics.
TRIALSCOPE — A Framework for Clinical Trial Simulation from Real-World Data [4]	2025	Presents a complete computational framework to simulate clinical trials using real-world data. It does not limit itself to evaluating models but defines a structured, modular, and reproducible procedure.	Provides a validation testing suite applicable to a comprehensive framework, robust quantitative metrics, and a methodological solution consisting of five tests to detect systematic errors.	Simulates trials using real-world data, testing multiple clinical scenarios with real variability in eligibility criteria and patient characteristics.
MedAgentBench: A Virtual EHR Environment to Benchmark Medical LLM Agents [6]	2025	Belongs to this category because it creates a virtual operational environment that enables evaluation of LLM-based medical agents across multiple clinical tasks with defined tasks, scenarios, and rules.	Virtual EHR environment with 100 simulated patients, 700,000 longitudinal entries, 300 clinical tasks, an interaction engine for EHR navigation and decision-making. Defines rules, constraints, and operational workflows for clinical agents.	Provides an evaluation benchmark oriented toward clinical agents capable of assessing clinical reasoning, EHR navigation, and operational performance. Establishes comparable metrics for complex tasks.
LLM-Based Medical Document Evaluation: Integrating Human Expert Insights [11]	2025	Proposes a hybrid methodological framework to evaluate medical documents generated by LLMs, combining automated evaluation with structured expert review. Defines criteria, steps, and evaluation mechanisms.	Proposes a three-module framework (Qualitative, Quantitative-Qualitative, and Expert Knowledge-Integrated) based on Chain of Thought (CoT) reasoning to evaluate medical documents generated by LLMs.	Formalizes human expert participation in generative model evaluation, defining protocols, scales, and reconciliation mechanisms that enable reproducible and auditable evaluations.

Continued on next page

Article Title	Year	Reason for Inclusion	Architectures and Operational Frameworks	Innovation and Methodological Contribution
A GEN AI Framework for Medical Note Generation [7]	2024	Proposes an integrated system (MediNotes) that combines Automatic Speech Recognition (ASR),Retrieval-Augmented Generation (RAG), and LLMs, with quantitative evaluation (Recall-Oriented Understudy for Gisting Evaluation (ROUGE), BERTScore,BERT-based Learned Evaluation for Text Generation (BLEURT)) and real clinical validation.	Multimodal architecture: ASR module for transcription, RAG for contextual integration, generative LLM for note production, traceability mechanisms, clinical audit, and human validation.	Integrates ASR + RAG + LLM into an operational clinical system; establishes a replicable and validated workflow for automated clinical note generation.
Enhanced Pre-Recruitment Framework for Clinical Trial Questionnaires Through the Integration of Large Language Models and Knowledge Graphs [16]	2025	Included because it defines a hybrid framework combining LLMs with knowledge graphs to preselect candidates for clinical trials. Proposes reproducible rules, modules, and procedures.	Hybrid architecture: semantic extraction via LLM, structured reasoning with Knowledge Graphs, validation of clinical criteria, and a rule-based matching engine based on explicit rules and semantic relationships.	The innovation lies in integrating unstructured reasoning with formal structures,Knowledge Graph (KG), providing a reproducible workflow for candidate matching based on free medical text.
A Knowledge-Enhanced Two-Stage Generative Framework for Medical Dialogue Information Extraction [5]	2024	Belongs to this category because it presents a two-stage generative workflow for information extraction in physician–patient dialogues.	Stage 1: Preliminary generation for dialogue reconstruction. Stage 2: Supervised refinement with biomedical knowledge integration.	Contributes a staged methodology that improves accuracy in clinical extraction tasks in dialogues. Proposes reproducible and generalizable steps to other conversational contexts.
A Framework for Human Evaluation of Large Language Models in Healthcare [12]	2024	Included because it presents a standardized framework for human evaluation of LLMs in healthcare, defining instruments, rubrics, and annotation processes applicable through three phases: Planning, Implementation and Adjudication, and Scoring and Review.	Based on four dimensions: Quality, Utility, Explainability, and Safety. Includes evaluator guidelines, detailed criteria, inter-annotator agreement analysis, and discrepancy resolution workflow.	Systematizes human evaluation of LLMs, creating a comparable procedure across institutions.
A Framework to Assess Clinical Safety and Hallucination Rates of LLMs for Medical Text Summarisation [2]	2025	Classified here because it presents a formal framework to measure clinical safety, hallucinations, and risk in medical summaries.	Defines a taxonomy of hallucinations, clinical severity criteria, human audit pipeline, structured review, and comparative metrics across models.	Introduces explicit clinical risk metrics and systematic procedures to evaluate hallucinations in generative clinical models.

Continued on next page

Article Title	Year	Reason for Inclusion	Architectures and Operational Frameworks	Innovation and Methodological Contribution
Zero-Shot Learning to Extract Assessment Criteria and Medical Services from Preventive Healthcare Guidelines Using Large Language Models [8]	2024	Develops a zero-shot learning framework to extract clinical criteria and preventive services from medical guidelines using LLMs.	Structured prompting, zero-shot extraction, rule-based clinical filtering, and final manual validation by specialists.	Demonstrates that regulatory processes can be automated through a reproducible zero-shot workflow.
Towards Evaluating and Building Versatile Large Language Models for Medicine (MedS-Bench / MedS-Ins) [15]	2025	Classified here because it defines a multifunctional benchmark to evaluate CFMs across multiple clinical tasks, providing a complete methodological environment.	The framework integrates multiple tasks (Question Answering (QA), summarization, information extraction, fact verification) and standardized metrics (Accuracy, Bilingual Evaluation Understudy (BLEU),ROUGE, F1), consolidating multidimensional clinical performance evaluation.	Offers a reproducible and open framework useful for strengthening medical AI evaluation using real data.

Table S2: Studies evaluating technical performance and clinical applicability of clinical foundation models (RQ2).

Article Title	Year	Reason for Inclusion in Performance and Applicability	Clinical Performance	Applicability
A Novel Playbook for Pragmatic Trial Operations to Monitor and Evaluate Ambient Artificial Intelligence in Clinical Practice [1]	2024	Longitudinal evaluation of real clinical AI systems in hospital environments, measuring performance, real-world use, errors, and achievement of clinical objectives.	Evaluates operational performance in real clinical practice: error rates, model stability, quality of assisted decisions, and variability in clinical staff usage.	Designed to scale across clinical environments, integrating AI into EHR systems (Fast Healthcare Interoperability Resources (FHIR)-based, Epic) with continuous Plan–Do–Study–Act (PDSA) cycles.
The Shaky Foundations of Large Language Models and Foundation Models for Electronic Health Records [14]	2023	Critically analyzes real CFM performance in extraction, reasoning, and EHR coherence tasks; questions validity of common metrics and calls for calibration and impact-based metrics.	Identifies inconsistencies, high-risk hallucinations, diagnostic errors, lack of robustness to contextual variation, and systematic biases.	Demonstrates that CFMs are unsafe without human supervision and lack interoperability across hospital systems.
Large Language Model–Based Assessment of Clinical Reasoning Documentation in the Electronic Health Record Across Two Institutions [10]	2025	Evaluates LLM capability to analyze and score clinical reasoning in real notes across two institutions with cross-validation and expert review.	Measures diagnostic hypotheses, therapeutic plans, red flags, internal coherence, and narrative clarity.	Supports quality auditing of clinical documentation and detection of reasoning deficiencies.

Continued on next page

Article Title	Year	Reason for Inclusion in Performance and Applicability	Clinical Performance	Applicability
LLM-Based Medical Document Evaluation: Integrating Human Expert Insights [11]	2025	Proposes a three-module framework integrating automated metrics and structured expert review for medical document evaluation.	Uses ROUGE, BERTScore,BLEURT, and clinical rubrics: completeness, accuracy, clinical risk, and guideline concordance.	Integrable into documentation review workflows, audits, and assisted clinical record generation.
A GEN AI Framework for Medical Note Generation [7]	2024	Provides a reproducible multimodal architecture (ASR+RAG+LLM) with automated metrics and real clinical validation.	Evaluates transcription accuracy, note completeness, contextual coherence, guideline adherence, and error reduction.	Applicable to clinical note-taking, reducing documentation time and improving record quality.
A Knowledge-Enhanced Two-Stage Generative Framework for Medical Dialogue Information Extraction [5]	2024	Evaluates information extraction performance in physician–patient dialogues.	Measures accuracy, F1-score, completeness in extraction of symptoms, history, and responses compared to baseline models.	Applicable in telemedicine, triage systems, and automated interview documentation.
A Framework for Human Evaluation of Large Language Models in Healthcare [12]	2024	Standardized human-based evaluation of LLM outputs under criteria of utility, clarity, reasoning, safety, and clinical coherence.	Identifies recurrent clinical error patterns and reasoning limitations through structured human scoring.	Supports validation before clinical deployment and regulatory compliance.
A Framework to Assess Clinical Safety and Hallucination Rates of LLMs for Medical Text Summarisation [2]	2025	Direct evaluation of safety and hallucination rates in medical summaries generated by LLMs.	Measures omission, commission, distortion errors, clinical severity levels, and potential patient harm.	Establishes safety standards for EHR-integrated summarization systems.
Zero-Shot Learning to Extract Assessment Criteria from Preventive Healthcare Guidelines [8]	2024	Evaluates zero-shot clinical extraction of regulatory criteria using LLMs.	Measures precision, guideline adherence, regulatory concordance, and reduction of manual errors.	Applicable to regulatory automation and clinical coding processes.
MedAgentBench: A Virtual EHR Environment to Benchmark Medical LLM Agents [6]	2025	Evaluates LLM-based clinical agents in longitudinal simulated EHR tasks.	Measures diagnostic accuracy, care plan completeness, risk recognition, and navigation across longitudinal data.	Potential for advanced digital assistants and multidimensional decision support.
Towards Evaluating and Building Versatile Large Language Models for Medicine [15]	2025	Multifunctional evaluation benchmark across standardized medical tasks.	Assesses QA, reasoning, extraction, generation, guideline comprehension, and mixed-task performance with standardized metrics.	Enables model adaptation across modalities without significant performance loss.
Enhanced Pre-Recruitment Framework for Clinical Trial Questionnaires Through LLMs and Knowledge Graphs [16]	2025	Evaluates hybrid LLM+ KG system for automated pre-screening in clinical trials.	Measures extraction accuracy and eligibility classification performance.	Applicable to automated recruitment and regulatory processes.

Continued on next page

Article Title	Year	Reason for Inclusion in Performance and Applicability	Clinical Performance	Applicability
Applications and Future Prospects of Medical LLMs: A Survey Based on the M-KAT Conceptual Framework [3]	2024	Evaluates medical LLM performance across multiple clinical tasks through the M-KAT conceptual framework.	Documents reasoning performance, documentation generation quality, entity extraction accuracy, guideline adherence, hallucination patterns, and domain variability.	Identifies tasks benefiting from LLMs and those requiring intensive supervision.

Table S3: Studies addressing ethical, regulatory, and institutional safety considerations of clinical foundation models (RQ3)

Article Title	Year	Reason for Inclusion in Ethics and Safety	Risks and Safety	Ethical Considerations
A Novel Playbook for Pragmatic Trial Operations to Monitor and Evaluate Ambient Artificial Intelligence in Clinical Practice [1]	2024	Introduces a formal framework incorporating regulatory mechanisms, risk mitigation, human oversight, and continuous monitoring of clinical AI.	Establishes continuous monitoring procedures to detect errors, operational biases, EHR integration failures, and unintended clinical effects.	Proposes clear responsibilities, user participation, algorithmic transparency, human-in-the-loop (HITL) oversight, and institutional impact control.
TRIALSCOPE — A Framework for Clinical Trial Simulation from Real-World Data [4]	2025	Addresses regulatory validity of clinical simulations and ethical controls when using real-world data.	Evaluates risks of erroneous cohort inference, automated selection bias, generalization issues, and misinterpretation of regulated clinical criteria.	Includes data protection, transparency in synthetic cohort generation, and institutional responsibility in automated simulations.
The Shaky Foundations of Large Language Models and Foundation Models for Electronic Health Records [14]	2023	Critically examines structural limitations, risks, biases, and reliability failures in CFMs applied to EHRs.	Identifies hallucinations, diagnostic errors, systematic bias, adversarial vulnerability, and lack of traceability.	Discusses moral responsibility, patient harm risk, inequities from bias, and opacity in high-risk clinical contexts.
Large Language Model–Based Assessment of Clinical Reasoning Documentation in the Electronic Health Record Across Two Institutions [10]	2025	Evaluates risks associated with automated assessment of clinical reasoning.	Risk of misclassification of reasoning quality, erroneous penalization of clinicians, and overreliance on automation.	Emphasizes human supervision, transparency in evaluation criteria, and institutional accountability.
A Framework to Assess Clinical Safety and Hallucination Rates of LLMs for Medical Text Summarisation [2]	2025	Directly evaluates clinical risks and hallucinations in medical summarization.	Defines hallucination taxonomy, severity levels, and potential patient harm.	Discusses regulatory standards, disciplinary responsibility, and safety principles in generative systems.
Medical Ethics of Large Language Models in Medicine [9]	2024	Focuses entirely on ethical principles in medical LLM use.	Identifies bias amplification, vulnerability in marginalized populations, opacity, and systemic errors.	Analyzes beneficence, non-maleficence, autonomy, justice, and professional responsibility.

Continued on next page

Article Title	Year	Reason for Inclusion in Ethics and Safety	Risks and Safety	Ethical Considerations
It Takes More Than Enthusiasm: The Missing Infrastructure to Unlock AI's Potential in Medical Education [13]	2025	Discusses institutional and governance risks in AI adoption.	Highlights lack of auditing, absence of standards, misinformation risk, and regulatory gaps.	Calls for governance policies, accountability mechanisms, and educational quality control.
MedAgentBench: A Virtual EHR Environment to Benchmark Medical LLM Agents [6]	2025	Includes in-depth analysis of risks in automated clinical agent decisions.	Identifies navigation errors, inappropriate decisions, omission of critical data, and longitudinal reasoning failures.	Emphasizes need for human oversight, multicenter validation, regulatory safeguards, and institutional responsibility.
Enhanced Pre-Recruitment Framework for Clinical Trial Questionnaires Through LLMs and Knowledge Graphs [16]	2025	Addresses ethical risks in automated patient selection for clinical trials.	Risk of inappropriate exclusion or inclusion, structural bias, and semantic matching errors.	Considers recruitment equity, justice in representation, institutional responsibility, and transparency in selection criteria.
Applications and Future Prospects of Medical LLMs: A Survey Based on the M-KAT Conceptual Framework [3]	2024	Explicitly analyzes risks, ethical principles, and regulatory challenges in medical LLM implementation.	Identifies systemic risks, overgeneralization, cultural bias, recurrent clinical errors, and governance limitations.	Addresses algorithmic justice, transparency, explainability, clinical accountability, and regulatory needs.
A Framework for Human Evaluation of Large Language Models in Healthcare [12]	2025	Designed to evaluate safety, reliability, and quality of LLM outputs from a human-centered perspective.	Defines explicit safety dimensions and structured identification of clinical risk.	Incorporates meaningful human oversight, shared responsibility, transparency, and fairness in subgroup evaluation.

References

[1] Majid Afshar, Felice Resnik, Mary Ryan Baumann, Josie Hintzke, Kayla Lemmon, Anne Gravel Sullivan, Tina Shah, Anthony Stordalen, Michael Oberst, Jason Dambach, Leigh Ann Mrotek, Mariah Quinn, Kirsten Abramson, Peter Kleinschmidt, Tom Brazelton, Heidi Twedt, David Kunstman, Graham Wills, John Long, Brian W. Patterson, Frank J. Liao, Stacy Rasmussen, Elizabeth Burnside, Cherodeep Goswami, and Joel E. Gordon. A Novel Playbook for Pragmatic Trial Operations to Monitor and Evaluate Ambient Artificial Intelligence in Clinical Practice. *NEJM AI*, 2(9), August 2025. ISSN 2836-9386. doi: 10.1056/AIdbp2401267. URL https://ai.nejm.org/doi/10.1056/AIdbp2401267.

[2] Elham Asgari, Nina Montaña-Brown, Magda Dubois, Saleh Khalil, Jasmine Balloch, Joshua Au Yeung, and Dominic Pimenta. A framework to assess clinical safety and hallucination rates of LLMs for medical text summarisation. *npj Digital Medicine*, 8(1):274, May 2025. ISSN 2398-6352. doi: 10.1038/s41746-025-01670-7. URL https://www.nature.com/articles/s41746-025-01670-7.

[3] Ying Chang, Jian-ming Yin, Jian-min Li, Chang Liu, Ling-yong Cao, and Shu-yuan Lin. Applications and Future Prospects of Medical LLMs: A Survey Based on the M-KAT Conceptual Framework. *Journal of Medical Systems*, 48(1):112, December 2024. ISSN 1573-689X. doi: 10.1007/s10916-024-02132-5. URL https://link.springer.com/10.1007/s10916-024-02132-5.

[4] Javier González, Risa Ueno, Cliff Wong, Zelalem Gero, Jaspreet Bagga, Isabel Chien, Eduard Oravkin, Emre Kiciman, Aditya Nori, Roshanthi Weerasinghe, Rom S. Leidner, Brian Piening, Tristan Naumann, Carlo Bifulco, and Hoifung Poon. TRIALSCOPE — A Framework for Clinical Trial Simulation from Real-World Data. *NEJM AI*, 2(10), September 2025. ISSN 2836-9386. doi: 10.1056/AIoa2400859. URL https://ai.nejm.org/doi/10.1056/AIoa2400859.

[5] Zefa Hu, Ziyi Ni, Jing Shi, Shuang Xu, and Bo Xu. A Knowledge-enhanced Two-stage Generative Framework for Medical Dialogue Information Extraction. *Machine Intelligence Research*, 21(1):153–168, February 2024. ISSN 2731-538X, 2731-5398. doi: 10.1007/s11633-023-1461-5. URL https://link.springer.com/10.1007/s11633-023-1461-5.

[6] Yixing Jiang, Kameron C. Black, Gloria Geng, Danny Park, James Zou, Andrew Y. Ng, and Jonathan H. Chen. MedAgentBench: A Virtual EHR Environment to Benchmark Medical LLM Agents. *NEJM AI*, 2(9), August 2025. ISSN 2836-9386. doi: 10.1056/AIdbp2500144. URL https://ai.nejm.org/doi/10.1056/AIdbp2500144.

[7] Hui Yi Leong, Yifan Gao, and Shuai Ji. A GEN AI Framework for Medical Note Generation. In *2024 6th International Conference on Artificial Intelligence and Computer Applications (ICAICA)*, pages 423–429, Dalian, China, November 2024. IEEE. ISBN 979-8-3503-7725-5. doi: 10.1109/ICAICA63239.2024.10823004. URL https://ieeexplore.ieee.org/document/10823004/.

[8] Xiao Luo, Fattah Muhammad Tahabi, Tressica Marc, Laura Ann Haunert, and Susan Storey. Zero-shot learning to extract assessment criteria and medical services from the preventive healthcare guidelines using large language models. *Journal of the American Medical Informatics Association*, 31(8):1743–1753, August 2024. ISSN 1067-5027, 1527-974X. doi: 10.1093/jamia/ocae145. URL https://academic.oup.com/jamia/article/31/8/1743/7696537.

[9] Jasmine Chiat Ling Ong, Shelley Yin-Hsi Chang, Wasswa William, Atul J. Butte, Nigam H. Shah, Lita Sui Tjien Chew, Nan Liu, Finale Doshi-Velez, Wei Lu, Julian Savulescu, and Daniel Shu Wei Ting. Medical Ethics of Large Language Models in Medicine. *NEJM AI*, 1(7), June 2024. ISSN 2836-9386. doi: 10.1056/AIra2400038. URL https://ai.nejm.org/doi/10.1056/AIra2400038.

[10] Verity Schaye, David DiTullio, Benedict Vincent Guzman, Scott Vennemeyer, Hanniel Shih, Ilan Reinstein, Danielle E Weber, Abbie Goodman, Danny T Y Wu, Daniel J Sartori, Sally A Santen, Larry Gruppen, Yindalon Aphinyanaphongs, and Jesse Burk-Rafel. Large Language Model–Based Assessment of Clinical Reasoning Documentation in the Electronic Health Record Across Two Institutions: Development and Validation Study. *Journal of Medical Internet Research*, 27:e67967, March 2025. ISSN 1438-8871. doi: 10.2196/67967. URL https://www.jmir.org/2025/1/e67967.

[11] Junhyuk Seo, Dasol Choi, Wonchul Cha, and Taerim Kim. LLM-Based Medical Document Evaluation: Integrating Human Expert Insights. In Mowafa S. Househ, Zain Ul Abideen Tariq, Mahmood Al-Zubaidi, Uzair Shah, and Elaine Huesing, editors, *Studies in Health Technology and Informatics*. IOS Press,

August 2025. ISBN 978-1-64368-608-0. doi: 10.3233/SHTI250995. URL https://ebooks.iospress.nl/doi/10.3233/SHTI250995.

[12] Thomas Yu Chow Tam, Sonish Sivarajkumar, Sumit Kapoor, Alisa V. Stolyar, Katelyn Polanska, Karleigh R. McCarthy, Hunter Osterhoudt, Xizhi Wu, Shyam Visweswaran, Sunyang Fu, Piyush Mathur, Giovanni E. Cacciamani, Cong Sun, Yifan Peng, and Yanshan Wang. A framework for human evaluation of large language models in healthcare derived from literature review. *npj Digital Medicine*, 7(1):258, September 2024. ISSN 2398-6352. doi: 10.1038/s41746-024-01258-7. URL https://www.nature.com/articles/s41746-024-01258-7.

[13] Laurah Turner, Christine Zhou, and Jesse Burk-Rafel. It Takes More Than Enthusiasm: The Missing Infrastructure to Unlock AI's Potential in Medical Education. *Academic Medicine*, 100(9S):S34–S38, September 2025. ISSN 1040-2446. doi: 10.1097/ACM.0000000000006104. URL https://journals.lww.com/10.1097/ACM.0000000000006104.

[14] Michael Wornow, Yizhe Xu, Rahul Thapa, Birju Patel, Ethan Steinberg, Scott Fleming, Michael A. Pfeffer, Jason Fries, and Nigam H. Shah. The shaky foundations of large language models and foundation models for electronic health records. *npj Digital Medicine*, 6(1):135, July 2023. ISSN 2398-6352. doi: 10.1038/s41746-023-00879-8. URL https://www.nature.com/articles/s41746-023-00879-8.

[15] Chaoyi Wu, Pengcheng Qiu, Jinxin Liu, Hongfei Gu, Na Li, Ya Zhang, Yanfeng Wang, and Weidi Xie. Towards evaluating and building versatile large language models for medicine. *npj Digital Medicine*, 8(1):58, January 2025. ISSN 2398-6352. doi: 10.1038/s41746-024-01390-4. URL https://www.nature.com/articles/s41746-024-01390-4.

[16] Chen Zihang, Liu Liang, Su Qianmin, Cheng Gaoyi, Huang Jihan, and Li Ying. Enhanced pre-recruitment framework for clinical trial questionnaires through the integration of large language models and knowledge graphs. *Scientific Reports*, 15(1):27398, July 2025. ISSN 2045-2322. doi: 10.1038/s41598-025-11876-0. URL https://www.nature.com/articles/s41598-025-11876-0.
